# Romanian Wild-Growing *Armoracia rusticana* L.—Untargeted Low-Molecular Metabolomic Approach to a Potential Antitumoral Phyto-Carrier System Based on Kaolinite

**DOI:** 10.3390/antiox12061268

**Published:** 2023-06-13

**Authors:** Adina-Elena Segneanu, Gabriela Vlase, Liviu Chirigiu, Daniel Dumitru Herea, Maria-Alexandra Pricop, Patricia-Aida Saracin, Ștefania Eliza Tanasie

**Affiliations:** 1 Institute for Advanced Environmental Research, West University of Timisoara (ICAM-WUT), Oituz nr. 4, 300086 Timisoara, Romania; gabriela.vlase@e-uvt.ro; 2 Research Center for Thermal Analysis in in Environmental Problems, West University of Timisoara, Pestalozzi St. 16, 300115 Timisoara, Romania; 3 Faculty of Pharmacy, University of Medicine and Pharmacy Craiova, 2, Petru Rareș, 200349 Craiova, Romania; liviu.chirigiu@umfcv.ro (L.C.); ada_patricia62@yahoo.com (P.-A.S.); eliza_tanasie@yahoo.com (Ș.E.T.); 4 National Institute of Research and Development for Technical Physics, 47 Mangeron Blvd, 700050 Iasi, Romania; dherea@phys-iasi.ro; 5 OncoGen Centre, Clinical County Hospital “Pius Branzeu”, Blvd. Liviu Rebreanu 156, 300723 Timisoara, Romania; alexandra.pricop@oncogen.ro

**Keywords:** secondary metabolites, horseradish, mass spectra, kaolinite, phyto-carrier system, antioxidant activity

## Abstract

Horseradish is a globally well-known and appreciated medicinal and aromatic plant. The health benefits of this plant have been appreciated in traditional European medicine since ancient times. Various studies have investigated the remarkable phytotherapeutic properties of horseradish and its aromatic profile. However, relatively few studies have been conducted on Romanian horseradish, and they mainly refer to the ethnomedicinal or dietary uses of the plant. This study reports the first complete low-molecular-weight metabolite profile of Romanian wild-grown horseradish. A total of ninety metabolites were identified in mass spectra (MS)-positive mode from nine secondary metabolite categories (glucosilates, fatty acids, isothiocyanates, amino acids, phenolic acids, flavonoids, terpenoids, coumarins, and miscellaneous). In addition, the biological activity of each class of phytoconstituents was discussed. Furthermore, the development of a simple target phyto-carrier system that collectively exploits the bioactive properties of horseradish and kaolinite is reported. An extensive characterization (FT-IR, XRD, DLS, SEM, EDS, and zeta potential) was performed to investigate the morpho-structural properties of this new phyto-carrier system. The antioxidant activity was evaluated using a combination of three in vitro, non-competitive methods (total phenolic assay, 2,2-Diphenyl-1-picrylhydrazyl (DPPH) radical-scavenging assay, and phosphomolybdate (total antioxidant capacity)). The antioxidant assessment indicated the stronger antioxidant properties of the new phyto-carrier system compared with its components (horseradish and kaolinite). The collective results are relevant to the theoretical development of novel antioxidant agent fields with potential applications on antitumoral therapeutic platforms.

## 1. Introduction

*Armoracia rusticana G. Gaertn.*, *B. Mey*. & *Scherb* (*Armoracia rusticana* L.) from the *Brassicaceae* family has been part of traditional European medicine since ancient times. The first mention of the healing effects of this plant (analgesic, diuretic, and antiparasitic) occurs in *De Materia Medica* [1]. Dacian medicine recommends horseradish as an anti-inflammatory cure for colds, coughs, and migraines [1]. Currently, horseradish root is used globally and on a large scale in food, food preservation, and traditional medicine [1].

It is known that there is an interdependence between the content of phytoconstituents in horseradish and different abiotic factors (pH, humidity, temperature, light, etc.) [2,3]. Furthermore, various studies reported that the profiles of metabolites considered responsible for the aroma of horseradish differ, depending on the genotype and plant maturity [3,4].

Recent research has shown that horseradish has collective therapeutic properties: antimicrobial, antifungal, anti-inflammatory, antiviral, and antitumor activity [5,6,7,8]. This herb’s notable pharmacological activity is due to the combined and synergistic action of its numerous secondary metabolites: glucosinolates, isothiocyanates, organo-sulfur compounds, flavonoids, terpenoids, phenolic acids, coumarins, amino acids, and fatty acids [6,7,8,9,10].

Recently, particular consideration has been given to advanced materials based on natural compounds, which feature extended-release, site-specific delivery and outperform the alternatives in terms of therapeutic activity (anti-tumor, antioxidant, antiviral, antimicrobial, neuroprotective, and anti-inflammatory) [11]. Various studies have investigated the isolation of glucosinolates, isothiocyanates, and organo-sulfur compounds, the main bioactive compounds of horseradish. It has been reported that their chemical stability and implicit bioavailability are influenced by time and temperature [12].

Among the foremost challenges related to new drug discovery from natural products are the composition and proportion differences of secondary metabolites resulting from the influence of biotic and abiotic factors [11,13]. Furthermore, the total synthesis of some phytoconstituents with sophisticated chemical structures and numerous chiral centers is demanding [14]. 

The use of natural products, especially those based on medicinal plants, has seen an upward trend across the whole world in recent years [15,16,17]. Although, for the majority of the population, herbal medicine is the primary strategy used in various ailments, recently, particularly in developed countries, natural products have begun to take an increasingly important place in modern civilization due to their high level of biocompatibility and weak side effects [15,16,17].

Several studies have reported the possible toxic effects of the different herbal products available on the market, which are mainly due to self-administration and exceeding the dosage [15,16,17]. The pandemic also contributed to this situation, and numerous deaths and severe complications were registered due to the effective lack of medicines [15,16,17]. Therefore, the most recent studies address the development of new plant-based materials with high performance, binding-site specificity, and controlled release [11,13]. Particular consideration is given to secondary metabolites with high levels of antioxidant, antimicrobial, antiviral, anti-inflammatory, neuroprotective, and antitumor activity [11].

On the other hand, clay therapeutic, food, and protective applications are an integral parts of human culture [18]. In Mesopotamia, Ancient Egypt, and Ancient Greece, clay was used for its anti-inflammatory, antiseptic, and wound-healing properties [18]. The great scholars of the ancient world, Hippocrates and Aristotle, were the first to create a classification of therapeutic clays according to their origin, chemical composition, and biological activity [18].

Recent studies have demonstrated that due to its outstanding physico-chemical properties, including small grain size (in the micrometric order), and large specific surface area (of approximately 100 m^2^/g), ensuring high adsorption, swelling, intercalation, and cation-exchange capacity, mineral clays can be used as carrier materials or drug-delivery-system substrates or supports [19,20,21].

In addition, various studies have confirmed the biological properties of clay minerals and reported high chemical stability and the absence of toxicity in vivo. Currently, clay mineral applications are used as active agents or excipients in numerous pharmaceutical and dermato-cosmetic preparations [19,20,21,22,23].

Kaolinite, Al_2_Si_2_O_5_(OH)_4_, with a ratio of SiO_2_ to Al_2_O_3_ of approximately 1.18:1, consists of a two-dimensional layer of silica groups linked to a layer of aluminum groups. The distance between the two layers is about 7.2 Å, and it has minor cation-exchange capacity. Furthermore, hydrogen bonds restrict the possibility of expansion or swelling between layers. The surface area is 10–30 m^2^/g. Due to its high chemical stability and inertness in vivo, kaolinite has numerous pharmaceutical applications (anti-inflammatory, antiviral, detoxification, hemostatic, antitumoral, protection against gastro-intestinal problems and skin damage, pelotherapy, detoxification, and others) [23,24,25,26].

Kaolinite increases the bioavailability of the drug through a controlled release and an oral administration route [23,25]. Many studies reported different drug-delivery systems based on clay minerals for use as in antioxidant, anti-inflammatory, antibiotic, antitumor, antimycotic, anticoagulant, antidiabetic, osteoporosis, and cardioprotective, applications, among others. The main benefits are the prolonged release, increased bioavailability, and minimized toxicity [24,25,27].

The most recent studies addressed the development of antitumoral and immunomodulation drug-delivery systems [24,25,27,28].

It is well-known that the excessive generation of reactive oxygen species (ROS) causes the onset of serious pathologies, including cancer [29,30,31,32]. Numerous studies investigated the use of antioxidants as a novel and potent approach to cancer prevention and treatment [29,30,31,32]. It is acknowledged that the excessive generation of reactive oxygen species (ROS) causes the onset of serious pathologies, including cancer [29,30,31,32]. Consequently, many studies have investigated the antioxidant function as a novel and robust approach to cancer prevention and treatment [29,30,31,32]. However, there are still many controversies regarding the effectiveness of antioxidants in cancer therapy [29,30,31,32]. Nevertheless, the most recent studies reported some possible factors that can significantly reduce their beneficial effects, such as low bioavailability and low transmembrane permeability, the absence of an adequate dosage, uneven distribution, and others [32].

Furthermore, the biological activity of a plant is the result of the synergistic action of the mixture or complex of secondary metabolites in different proportions [9,33,34].

The antioxidant activities of phytoconstituents are determined by several factors: diversity, climatic factors (temperature, humidity, pH, and soil chemical composition), and harvest maturity stage [35]. Additionally, antioxidant agents are grouped into several categories depending on their mechanism of action (direct or indirect), their source, and the physical-chemical properties of the biomolecule (size, solubility, and others) [36,37,38]. The efficiency of an antioxidant agent is influenced by several criteria: metabolism pathway, bioavailability, rate constant, concentration, the chemical structure of the biomolecule, and others [36,39].

The development of a successful phyto-carrier assembly relies upon the complementary and synergistic action of the carrier and the secondary metabolites. Furthermore the morpho-structural characteristics, chemical and thermal stability, and biological properties of the carrier have an essential role [13].

Consequently, the high-performance carrier system based on kaolinite development represents a novel multifunctional strategy that will overcome the limitations of the current therapeutic approach related to the drug resistance of cancer cells and ensure site-specific targeting and controlled release.

This study investigates, for the first time, the development and characterization of a phyto-engineered carrier system that accumulates the biological properties of horseradish and kaolinite. Furthermore, to the best of our knowledge, another novelty of this study is the identification of a complete low-molecular-weight metabolite profile of *Armoracia rusticana*, grown in the wild in Romania.

## 2. Materials and Methods

All used reagents were analytical grade. Methanol, chloroform, dichloromethane, and ethanol were acquired from Sigma-Aldrich (München, Germany) and used without further purification. The DPPH (2,2-diphenyl-1-picrylhydrazyl), β-carotene Type II, synthetic (≥95%), ascorbic acid, AgNO_3_, sodium citrate, sodium carbonate, Folin–Ciocalteu phenol reagent (2 N), potassium persulfate, sodium phosphate, ammonium molybdate, and potassium chloride of 99% purity or higher were purchased from Sigma-Aldrich (München, Germany). Propyl gallate (purum) was purchased from Fluka (Buchs, Switzerland). The horseradish sample (leaves (28 cm in height) and roots (lengths of about 35 cm) were collected in November 2022 from the area of Timis County, Romania (geographic coordinates 45°45′59.99″ N 21°17′60.00″ E) and taxonomically authenticated at the University of Medicine and Pharmacy Craiova, Romania. Kaolinite was purchased from local market in Timisoara, Romania. The double distilled water (DDW) was used throughout the experiments. 

### 2.1. Phyto-Carrier-System Components’ Preparation

#### 2.1.1. Plant-Sample Preparation for Chemical Screening 

The plant samples (roots and leaves) were cut and then quickly frozen in liquid nitrogen (180 °C). Subsequently, they were ground and sieved to obtain a particle size lower than 0.45 mm and then stored at −38 °C to prevent enzyme-mediated degradation of phytoconstituents, in a 100 mL conical flask containing 1.5 g dried plant sample and 15 mL of solvent (methanol/chloroform = 1:1). Subsequently, the mixture was subjected to sonication extraction for 30 min at 35 °C with a frequency of 60 kHz. The resulting solution was concentrated using a rotary evaporator, and the obtained residue was dissolved in 10 mL MeOH. The obtained extract was centrifuged (10,000 rot/min, 10 min), and the supernatant was filtered through a 0.2 µm syringe filter and stored at −25 °C until further analysis. All samples were prepared in triplicate.

#### 2.1.2. GC-MS Analysis

Gas chromatography was carried out on a GCMS-QP2020NX Shimadzu apparatus with a ZB-5MS capillary column (30 m × 0.25 mm id × 0.25 µm) (Agilent Technologies, Santa Clara, CA, USA), helium, flow of 1 mL/min. 

#### 2.1.3. GC–MS Separation Conditions

The oven-temperature program started from 50 °C to 300 °C with a rate of 5 °C/minute, and it was finally kept at this temperature for 3 min. The temperature of the injector was 280 °C and the temperature at the interface was 230 °C. The compounds’ mass was registered at 70 eV ionization energy starting after 3 min of solvent delay. The source of the mass spectrometer was heated at 235 °C and the MS quad was heated at 165 °C. The mass values of identified compounds were scanned from 50 amu to 570 amu. Compounds were identified based on their mass spectra, which were compared to the NIST0.2 mass-spectra-library database (USA National Institute of Science and Technology Software, (NIST, Gaithersburg, MD, USA). Furthermore, the calculated retention indices (RIs) for each compound were compared with the Adams indices in the literature (Table 1) [40].

#### 2.1.4. Mass Spectrometry

The MS experiments were performed using EIS-QTOF-MS (Bruker Daltonics, Bremen, Germany). The mass spectra were acquired in the positive ion mode in a mass range of 100–3000 *m*/*z*, scan speed was 2.0 scans/s, collision energy was 25–85 eV, and the temperature of source block was 80 °C. The identification of phytoconstituents was based on standard library NIST/NBS-3 (National Institute of Standards and Technology/National Bureau of Standards) (NIST, Gaithersburg, MD, USA). The obtained mass-spectra values and the identified secondary metabolites are presented in Table 2.

#### 2.1.5. Phyto- Carrier System Preparation

For each analysis, 2.5 g of sample was prepared from dried horseradish, and kaolinite powder was added (horseradish/kaolinite nanoparticles = 1:3) at room temperature (22 °C), ground, and homogenized for 10 min using a pestle and mortar. 

### 2.2. Characterization of the Phyto- Carrier System

#### 2.2.1. Fourier-Transform Infrared (FTIR) Spectroscopy

Data collection was conducted after 20 recordings at a resolution of 4 cm^−1^, in the range of 4000–400 cm^−1^, on Shimadzu AIM-9000 with ATR devices (Shimadzu, Kyoto, Japan).

#### 2.2.2. XDR Spectroscopy

The X-ray powder diffraction (XRD) was performed using a Bruker AXS D8-Advance X-ray diffractometer (Bruker AXS GmbH, Karlsruhe, Germany) equipped with a rotating sample stage, Anton Paar TTK low-temperature cell (−180 °C ÷ 450 °C), high vacuum, inert atmosphere, relative humidity control, and Anton Paar TTK high-temperature cell (up to 1600 °C). The XRD patterns were compared with those from the ICDD Powder Diffraction Database (ICDD file 04-015-9120). The average crystallite size and the phase content were determined using the whole-pattern profile-fitting method (WPPF).

#### 2.2.3. Scanning-Electron Microscopy (SEM) 

The SEM micrographs were obtained with a SEM–EDS system (QUANTA INSPECT F50) equipped with a field-emission gun (FEG), 1.2 nm resolution, and energy-dispersive X-ray spectrometer (EDS) with a MnK resolution of 133 eV.

#### 2.2.4. Dynamic Light Scattering (DLS) Particle-Size-Distribution Analysis

The DLS analysis was carried on a Microtrac/Nanotrac 252 (Montgomeryville, PA, USA). Each sample was analyzed in triplicate at room temperature (22 °C) at a scattering angle of 172°.

#### 2.2.5. Zeta-Potential Analysis

The zeta-potential analysis was conducted using an AMERIGO particle-size and zeta-potential analyzer (Pessac, France), with six measurements/s. The main experimental conditions were as follows. Electrode distance: 5 mm; temperature: 25 °C; conductivity: 5.10 V; carrier frequency: 8210 Hz; reference intensity: 2660 kcps; applied field: 20.27 V/cm; and scattering intensity: 2850 kcps.

#### 2.2.6. Antioxidant Activity

The antioxidant activity of the newly phyto-carrier system was estimated using three different assays: a 2,2-diphenyl-1-picrylhydrazyl (DPPH) radical-scavenging assay, a Folin–Ciocalteu assay, and phosphomolybdate assay (total antioxidant capacity).

The phyto-carrier system (0.25 g) and horseradish (0.3 g) samples were dissolved in methanol (10 mL and 12 mL, respectively). The mixtures were stirred at room temperature (22 °C) for 8 h, and then centrifuged at 10,000 rpm for 10 min. The supernatant was then collected for use in the antioxidant assays (2,2-diphenyl-1-picrylhydrazyl (DPPH) radical-scavenging assay, Folin–Ciocalteu assay, and phosphomolybdate assay (total antioxidant capacity).

#### 2.2.7. Determination of Total Phenolic Content

The total phenolic contents in the newly phyto-carrier system and horseradish samples were determined spectrophotometrically according to the Folin–Ciocalteu procedure adapted from the literature [41].

A volume of 2 mL of Folin–Ciocalteu reagent (0.2 N) and 0.2 mL of each sample were vortexed and stored at room temperature (22 °C) for 8 min, in the dark. Sequentially, 2 mL sodium carbonate (7.5%) was added. Next, after two h of incubation at room temperature (vortexed in the dark) the absorbance was measured at 725 nm using a Tecan i-control, 1.10.4.0 infinite 200 Pro spectrophotometer with Corning 96 flat-bottomed clear polystyrol plates (Tecan, Männedorf, Switzerland). The phenol content was expressed in gallic acid equivalents (mg GAE/g sample) using a propyl gallate standard calibration curve between 1 mg/mL and 12.5 µg/mL in methanol [42].

Sample extract concentrations were calculated based on the linear equation obtained from the standard curve (y = 0.9873x − 0.0989).

#### 2.2.8. DPPH Radical-Scavenging Assay

The stock solution was prepared by dissolving 2 mg DPPH in 20 mL MeOH followed by dilutions for a calibration curve with a range of concentrations between 3.12 µg/mL and 0.1 mg/mL. Serial dilutions of ascorbic acid and β-carotene were used as positive standards and MeOH as a vehicle control sample. The ratio (*v*/*v*) of DPPH to samples was of 1:1. All samples were placed, in triplicate, in a 96-well plate and stored at 22 °C for 30 min in the dark. At 515 nm, the absorbance was determined on a Tecan i-control, 1.10.4.0 infinite 200 Pro spectrophotometer (Tecan Group Ltd., Männedorf, Switzerland). 

The obtained results were used to calculate the average and the inhibition percentage (Inh%) (Equation (1)).

Inh% = (A0 − As)/A0 × 100 (1)

where:

A0 = vehicle control absorbance;

As—sample absorbance.

Further, the IC_50_ value was obtained from the inhibition percentage using the equation of a calibration curve generated for each sample and standard. The results were presented as Inh% versus concentration (µg/mL) [43].

#### 2.2.9. Phosphomolybdate Assay (Total Antioxidant Capacity)

The total-antioxidant-capacity assay of the new phyto-carrier system and horseradish samples was carried out by the phosphomolybdenum procedure using ascorbic acid as standard [44]. 

A volume of 5 mL reagent solution (0.6 M sulfuric acid, 28 mM sodium phosphate and 4 mM ammonium molybdate) and 0.5 mL of each sample were placed into a water bath at 95 °C for 120 min. Next, the mixed solutions were cooled at room temperature (22 °C). The absorbance was measured at 765 nm using a UV-VIS Perkin-Elmer Lambda 35 (Perkin Elmer, Waltham, MA, USA).

A blank solution was used (5 mL reagent was added in 0.5 mL methanol, and then the mixture was incubated in the same experimental conditions (at 95 °C for 120 min, and then cooled at room temperature (22 °C)). Total antioxidant capacity was determined according to the following equation (Equation (2))

Total antioxidant capacity (%) = [(Abs. of control − Abs. of sample)/(Abs. of control] × 100(2)

The results are presented as μg/mL of ascorbic acid equivalents (AAE).

#### 2.2.10. Statistical Analysis

All results were obtained with Microsoft Office Excel 2019. Data were used to calculate the average of three replicates for all samples, and all calibration curves and concentrations.

## 3. Results and Discussion

Plants contain an extensive range of categories of secondary metabolites, with complex chemical compositions [45,46].

In recent years, numerous studies addressed the phytochemical composition and pharmacological activities of metabolites from horseradish roots [4,5,6,7,8,47,48,49,50,51,52,53,54]. There are relatively few studies related to the phytoconstituents from horseradish leaves [1,7,8,55].

Nevertheless, a specific plant’s biological activity is the synergistic action of whole phytoconstituent result. Furthermore, researchers have reported that various biotic or abiotic factors (stress, pathogens, and others) altered the metabolite balance and, implicitly, their variability and interrelation [56,57,58]. In addition, several other elements (the part of the plant used, the extraction process, and the solvent used) influence the type and proportion of bioactive compounds collected from plants [58,59,60,61]. Therefore, a plant extract’s pharmacological activity differs from the experimental conditions, making it difficult to evaluate the relationship between chemical composition and therapeutic effect [58]. 

The chemical screening of the phytoconstituents from the horseradish sample was carried out via gas chromatography coupled with mass spectroscopy (GC-MS) and electrospray ionization–quadrupole time-of-flight mass spectrometry (ESI–QTOF–MS) analysis.

The gas-chromatography method coupled with mass spectroscopy (GC–MS) is the most convenient technique for secondary metabolites with relatively low molecular mass (volatile compounds, fatty acids, etc.), providing efficient separation and identification [62]. 

**Figure 1:**
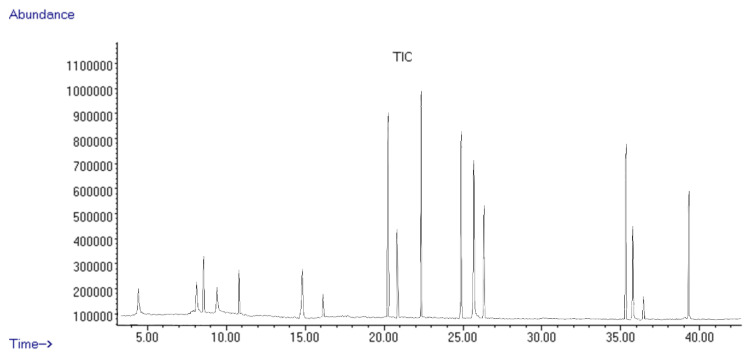
TIC chromatogram of horseradish extract.

The GC–MS analysis (Figure 1) revealed the separation of several low-molecular-weight metabolites from the horseradish sample.

The results are summarized in Table 1, which presents the tentative compound identification from the horseradish sample using GC–MS.

**Table 1:** Main compounds identified by GC–MS analysis of horseradish sample.

No	Retention Time (RT)	Retention Index (RI) Determined	Adams Indices (AI)	Area%	Compound Name	Ref
1	4.23	503	517	0.67	dimethyl sulfide	[47]
2	8.36	1005	1175	0.84	*α*-phellandrene	[7,48]
3	8.79	1387	1397	1.13	junipene	[7,48]
4	9.44	275	279	0.69	carbonyl sulphide	[7,48]
5	10.76	1169	1173	1.13	menthol	[7,48]
6	14.88	557	574	2.24	carbon disulfide	[7,48]
7	16.02	1198	1202	0.56	3-phenylpropionitrile	[47]
8	20.09	1713	1699	10.47	isobutyl isothiocyanate	[48]
9	20.97	652	706	4.76	2-ethylfuran	[63]
10	22.38	889	1349	13.82	allyl isothiocyanate	[48]
11	24.97	949	963	12.59	3-butenyl isothiocyanate	[48]
12	25.67	1075	1113	11.88	2-pentyl isothiocyanate	[48]
13	26.47	1287	1303	10.77	cyclopentyl isothiocyanate	[48]
14	35.08	1363	1317	9.74	benzylisothiocyanate	[7,48]
15	35.97	1317	1435	5.41	erucin	[64]
16	36.49	1215	1231	0.55	2-pentylfuran	[63]
17	39.37	1165	1267	7.88	phenylisothiocyanate	[48]

RI—retention indices calculated based upon a calibration curve of a C8–C20 alkane standard mixture.

The GC–MS analysis showed the presence of seventeen major components, accounting for 95.13% of the total peak area in the horseradish samples (Figure 1).

However, thermally unstable biomolecules require additional procedures (for instance, derivatization). Therefore, the mass-spectrometry method was selected for the metabolite-profile screening [65].

### 3.1. Mass-Spectrometric Analysis of Horseradish Sample

**Figure 2:**
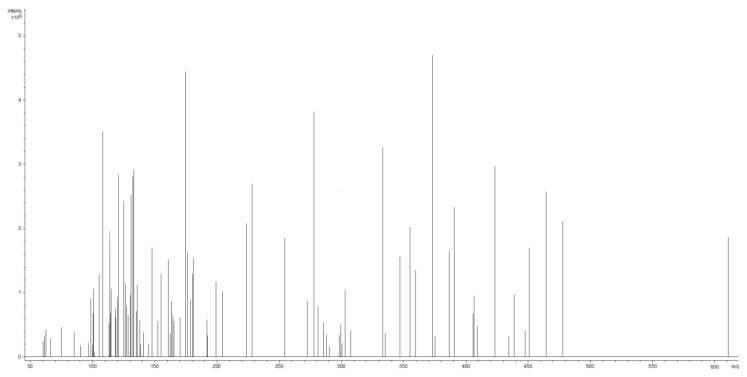
The mass spectrum of *Armoracia rusticana* L.

The spectra revealed a complex combination of low-molecular-weight components, of which some were detected. The mass spectra of the identified metabolites were compared with those of the NIST/EPA/NIH Mass Spectral Library 3.0 database, in addition to a literature review [7,48,55,66]. The mass spectrum and the phytoconstituents identified by the ESI–QTOF–MS analysis are presented in Figure 2 and Table 2, respectively.

**Table 2:** The molecules identified through electrospray-ionization–quadrupole time-of-flight mass spectrometry (ESI–QTOF–MS) analysis.

No	*m*/*z* Detected	Theoretic *m*/*z*	Formula	Tentative of Identification	Category	Ref
1	61.05	60.05	C_2_H_4_O_2_	acetic acid	organic acid	[48]
2	61.08	60.08	COS	carbonyl sulphide	sulfur compound	[48]
3	63.14	62.14	C_2_H_6_S	dimethyl sulfide	sulfur compound	[7]
4	68.09	67.09	C_4_H_5_N	allyl cyanide	miscellaneous	[48]
5	77.15	76.15	CS_2_	carbon disulfide	sulfur compound	[7]
6	87.11	86.13	C_5_H_10_O	pentanal	aldehyde	[7]
7	91.01	90.03	C_2_H_2_O_4_	oxalic acid	organic acid	[48]
8	97.11	96.13	C_6_H_8_O	2-ethylfuran	furans	[7]
9	99.13	98.14	C_6_H_10_O	3-hexenal	aldehyde	[49]
10	100.15	99.16	C_4_H_5_NS	allyl isothiocyanate	isothiocyanates	[7,48]
11	101.14	100.16	C_6_H_12_O	hexanal	aldehyde	[48]
12	103.13	102.13	C_5_H_10_O_2_	isovaleric acid	organic acid	[49]
13	104.11	103.12	C_4_H_9_NO_2_	γ-aminobutyric acid	organic acid	[50]
14	107.11	106.12	C_7_H_6_O	benzaldehyde	aldehyde	[48]
15	109.13	108.14	C_7_H_8_O	benzyl alcohol	organic acid	[48]
16	114.17	113.18	C_5_H_7_NS	3-butenyl isothiocyanate	isothiocyanates	[48]
17	116.11	115.13	C_5_H_9_NO_2_	proline	amino acid	[50]
18	116.18	115.20	C_5_H_9_NS	isobutyl isothiocyanate	isothiocyanates	[48]
19	117.06	116.07	C_4_H_4_O_4_	fumaric acid	organic acid	[48]
20	119.07	118.09	C_4_H_6_O_4_	succinic acid	organic acid	[48]
21	120.11	119.12	C_4_H_9_NO_3_	threonine	amino acid	[51]
22	121.14	120.15	C_8_H_8_O	phenylacetaldehyde	aldehyde	[49]
23	122.17	121.16	C_3_H_7_NO_2_S	cysteine	amino acid	[52]
24	127.19	126.20	C_8_H_14_O	vinyl amyl ketone	ketone	[66]
25	128.19	127.21	C_6_H_9_NS	cyclopentyl isothiocyanate	isothiocyanate	[48]
26	129.15	128.17	C_10_H_8_	naphthalene	miscellaneous	[7]
27	130.21	129.23	C_6_H_11_NS	2-pentyl isothiocyanate	isothiocyanate	[7,48]
28	132.19	131.17	C_9_H_9_N	3-phenylpropionitrile	miscellaneous	[7]
29	133.11	132.12	C_4_H_8_N_2_O_3_	asparagine	amino acid	[8]
30	135.07	134.09	C_4_H_6_O_5_	malic acid	organic acid	[56]
31	135.15	134.17	C_9_H_10_O	4-ethylbenzaldehyde	aldehyde	[7]
32	137.13	136.15	C_8_H_8_O_2_	anisaldehyde	aldehyde	[49]
33	137.25	136.23	C_10_H_16_	α-phellandrene	terpenoid	[56]
34	139.11	138.12	C_7_H_6_O_3_	p-salicylic acid	organic acid	[56]
35	139.19	138.21	C_9_H_14_O	2-pentylfuran	furans	[7]
36	143.21	142.24	C_9_H_18_O	nonanal	aldehyde	[7]
37	147.17	146.19	C_6_H_14_N_2_O_2_	lysine	amino acid	[50]
38	150.19	149.21	C_8_H_7_NS	benzyl isothiocyanate	isothiocyanate	[6,48,53,54,55]
39	153.13	152.15	C_8_H_8_O_3_	vanillin	aldehyde	[50]
40	157.25	156.26	C_10_H_20_O	menthol	terpenoid	[7]
41	162.23	161.3	C_6_H_11_NS_2_	erucin	isothiocyanate	[49]
42	164.21	163.24	C_9_H_9_NS	phenethyl isothiocyanate	isothiocyanate	[48]
43	165.15	164.16	C_9_H_8_O_3_	coumarinic acid	phenolic acid	[55]
44	166.21	165.19	C_9_H_11_NO_2_	phenylalanine	amino acid	[51]
45	167.23	166.22	C_9_H_14_N_2_O	2-sec-butyl-methoxy-pyrazine	miscellaneous	[49]
46	171.13	170.12	C_7_H_6_O_5_	gallic acid	phenolic acid	[50]
47	175.19	174.20	C_6_H_14_N_4_O_2_	arginine	amino acid	[50]
48	177.13	176.12	C_6_H_8_O_6_	ascorbic acid	organic acid	[55]
49	179.15	178.14	C_9_H_6_O_4_	esculetin	coumarin	[8]
50	181.15	180.16	C_9_H_8_O_4_	caffeic acid	phenolic acid	[50]
51	182.17	181.19	C_9_H_11_NO_3_	tyrosine	amino acid	[52]
52	193.11	192.12	C_6_H_8_O_7_	citric acid	organic acid	[55]
53	193.17	192.17	C_10_H_8_O_4_	scopoletin	coumarin	[8]
54	199.19	198.17	C_9_H_10_O_5_	syringic acid	phenolic acid	[55]
55	205.33	204.35	C_15_H_24_	junipene	terpenoid	[7]
56	225.19	224.21	C_11_H_12_O_5_	sinapinic acid	phenolic acid	[55]
57	229.36	228.37	C_14_H_28_O_2_	myristic acid	fatty acid	[50]
58	255.40	254.41	C_16_H_30_O_2_	13-hexadecenoic acid	fatty acid	[50]
59	273.43	272.42	C_16_H_32_O_3_	beta-hydroxypalmitic acid	fatty acid	[50]
60	279.39	278.4	C_18_H_30_O_2_	pinolenic acid	fatty acid	[50]
61	281.38	280.4	C_18_H_32_O_2_	linoleic acid	fatty acid	[6]
62	287.25	286.24	C_15_H_10_O_6_	kaempferol	flavonoid	[55]
63	289.41	288.42	C_16_H_32_O_4_	9,10-dihydroxypalmitic acid	fatty acid	[50]
64	291.25	290.27	C_15_H_14_O_6_	catechin	flavonoid	[55]
65	299.49	298.5	C_18_H_34_O_3_	17-hydroxyoleic acid	fatty acid	[50]
66	300.51	299.5	C_18_H_37_NO_2_	sphingosine	miscellaneous	[50]
67	301.49	300.5	C_18_H_36_O_3_	14-hydroxystearic acid	fatty acid	[50]
68	303.25	302.23	C_15_H_10_O_7_	quercetin	flavonoid	[66]
69	309.49	308.5	C_20_H_36_O_2_	eicosadienoic acid	fatty acid	[50]
70	317.51	316.5	C_18_H_36_O_4_	10,11-dihydroxy stearic acid	fatty acid	[50]
71	333.25	332.26	C_13_H_16_O_10_	glucogallin	tannin	[50]
72	334.31	333.3	C_8_H_15_NO_9_S_2_	glucocapparin	glucosinolates	[6]
73	348.39	347.4	C_9_H_17_NO_9_S_2_	glucolepidiin	glucosinolates	[6]
74	355.29	354.31	C_16_H_18_O_9_	chlorogenic acid	phenolic acid	[55]
75	360.39	359.4	C_10_H_17_NO_9_S_2_	sinigrin	glucosinolates	[8]
76	374.39	373.4	C_11_H_19_NO_9_S_2_	gluconapin	glucosinolates	[6,8]
77	376.41	375.4	C_11_H_21_NO_9_S_2_	glucocochlearin	glucosinolates	[67]
78	388.39	387.4	C_12_H_21_NO_9_S_2_	glucobrassicanapin	glucosinolates	[6,8]
79	392.41	391.4	C_11_H_21_NO_10_S_2_	glucoconringiin	glucosinolates	[7]
80	407.51	406.5	C_11_H_20_NO_9_S_3_	glucoiberverin	glucosinolates	[6,8]
81	408.49	407.5	C_11_H_21_NO_9_S_3_	glucosativin	glucosinolates	[7]
82	410.39	409.4	C_14_H_19_NO_9_S_2_	glucotropaeolin	glucosinolates	[6,8]
83	424.52	423.5	C_11_H_21_NO_10_S_3_	glucoiberin	glucosinolates	[6,8]
84	436.49	435.5	C_13_H_25_NO_9_S_3_	glucoberteroin	glucosinolates	[6,8]
85	440.51	439.5	C_11_H_21_NO_11_S_3_	glucocheirolin	glucosinolates	[6,8]
86	449.52	448.5	C_16_H_20_N_2_O_9_S_2_	glucobrassicin	glucosinolates	[7]
87	452.49	451.5	C_13_H_25_NO_10_S_3_	glucoalyssin	glucosinolates	[7]
88	465.52	464.5	C_16_H_20_N_2_O_10_S_2_	5-hydroxyglucobrassicin	glucosinolates	[7]
89	479.51	478.5	C_17_H_22_N_2_O_10_S_2_	4-methoxyglucobrassicin	glucosinolates	[7]
90	611.49	610.5	C_27_H_30_O_16_	rutin	flavonoid	[55]

The metabolite profile from the horseradish sample conducted through the GC–MS and mass spectroscopy corroborated the data reported in the literature [6,7,8,48,49,50,51,52,53,54,55,63,64,65].

### 3.2. Screening and Classification of the Differential Metabolites

The 90 secondary metabolites identified through mass spectroscopy were assigned to different chemical classes: glucosilates (18.9%), fatty acids (11.12%), isothiocyanates (8.9%), amino acids (8.9%), phenolic acids (6.67%), flavonoids (4.45%), terpenoids (3.34%), coumarins (2.23%), and miscellaneous. The assignment of the identified secondary metabolites into different chemical categories is presented in Table 3.

**Figure 3:**
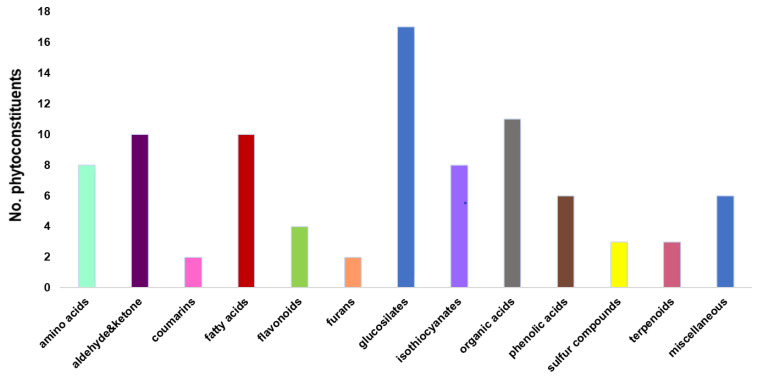
Phytoconstituent-classification bar chart for *Armoracia rusticana*.

Figure 3 presents the classification chart of the phytoconstituents from the horseradish sample based on the data analysis reported in Table 3.

According to Figure 3, *glucosinolates* are the largest category of phytochemicals, comprising about 19% of the total found in the horseradish sample. Recent studies demonstrated their antioxidant, anti-inflammatory, and antitumoral properties [7,66,67].

*Isothiocyanates* are a category of metabolites characteristic of cruciferous plants, with remarkable anti-cancer, anti-inflammatory, and neuroprotective effects [67,68].

Organo-sulfur phytoconstituents represented over 30% of all the metabolites identified in the horseradish sample. Various studies have shown that sulfur phytochemicals possess antioxidant, antiviral, antifungal, antibacterial, and antitumor properties [7,54,69,70].

*Fatty acids* represented more than 11% of the phytoconstituents identified in the horseradish sample. These secondary metabolites exhibit antioxidant, anti-inflammatory, cardio, and neuroprotective activities [71,72].

*Amino acids*: eight compounds were identified in the sample extract; the proportions of non-essential amino acids (proline, cysteine, asparagine, and tyrosine) and essential amino acids (threonine, lysine, phenylalanine, and arginine) were equal [73,74,75].

About one-third of the amino acids identified in the horseradish sample (arginine, phenylalanine, and proline) act as antitumor, neuroprotective, antiproliferative, and immunomodulating agents [71,74,75,76,77].

*Phenolic acids* are another class of phytochemicals with outstanding therapeutic properties (antioxidants, anti-inflammatory, antimicrobial, antidiabetic, antitumor, neuroprotective) [78,79].

The *Terpenoids* found in the horseradish samples were α-phellandrene, junipene, and menthol. Studies have reported that these have antitumor properties. Furthermore, menthol also acts as an antibacterial, antifungal, antipruritic, and analgesic agent [80,81,82].

*Flavonoids* are other category of secondary metabolites identified in the horseradish sample with notable pharmacological proprieties, including antioxidant, anti-inflammatory, antitumoral, and antimicrobial properties, as well as activities against neurodegenerative diseases (Alzheimer’s) [73,79,83]. 

The two *coumarins* identified in the horseradish sample, scopoletin and esculetin, show exceptional therapeutic activity, with antioxidant, anti-inflammatory, antitumor, hepatoprotective, and antidiabetic properties, as well as activities against neurodegenerative diseases (Alzheimer’s) [84,85].

Among the *miscellaneous* compounds identified in the horseradish sample, sphingosine exerts antitumoral, immunomodulatory, and neuroprotective activities [86,87,88]. Furthermore, glucogallin possesses antioxidant, anti-inflammatory, and antidiabetic properties [87]. 

**Figure 4:**
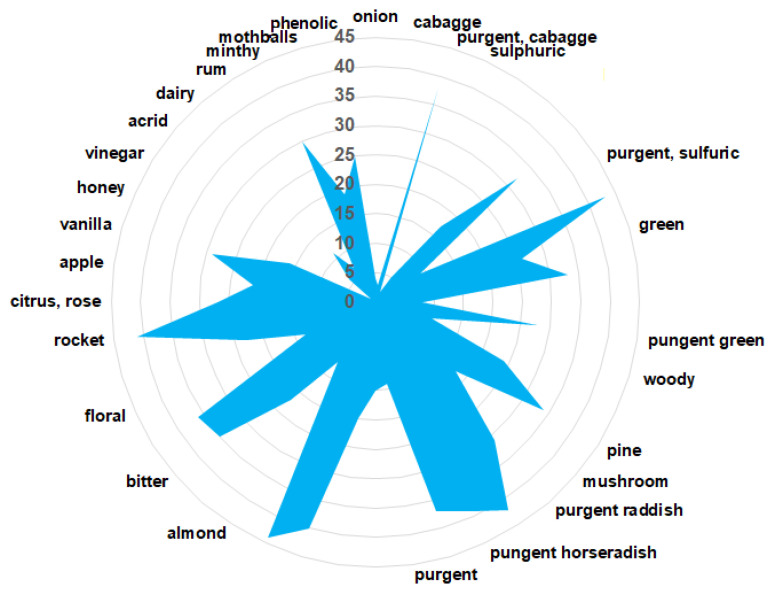
VOC-aroma profile of phytoconstituients identified in horseradish sample.

The aromatic compounds of volatile metabolites (VOCs) identified in the horseradish sample are shown in Table 4 and Figure 4. 

The predominant aromatic components of the investigated Romanian horseradish depend on different conditions (climatic conditions, maturity soil parameters, varieties, harvest time, and others) [4,7,47,48,49]. 

Their fragrances are unique, encompassing a purgent aroma with rocket and sulfuric, green, sweet-vanilla, and floral notes [4,7,47,48].

### 3.3. Phyto-Carrier System

The main challenges in the novel therapeutic approaches to cancer are the drug resistance of cancer cells, determined by the reduced retention interval, low permeability, the triggering of inactivation by the immune system, and the lack of specificity [89,90].

Hence, the development of an innovative phyto-carrier target system with cumulative and synergistic kaolinite and horseradish biological activity could make it possible to overcome the limitations related to vectorization, site-specific distribution, prolonged release, and membrane permeability.

### 3.4. FT-IR Spectroscopy

The use of FTIR is one of the most common analytical techniques, and it is considered fundamental in the analysis of complex carrier systems due to its features (sensitivity, flexibility, robustness, and specificity), allowing the investigation of interactions between biomolecules and mineral components [90].

**Figure 5:**
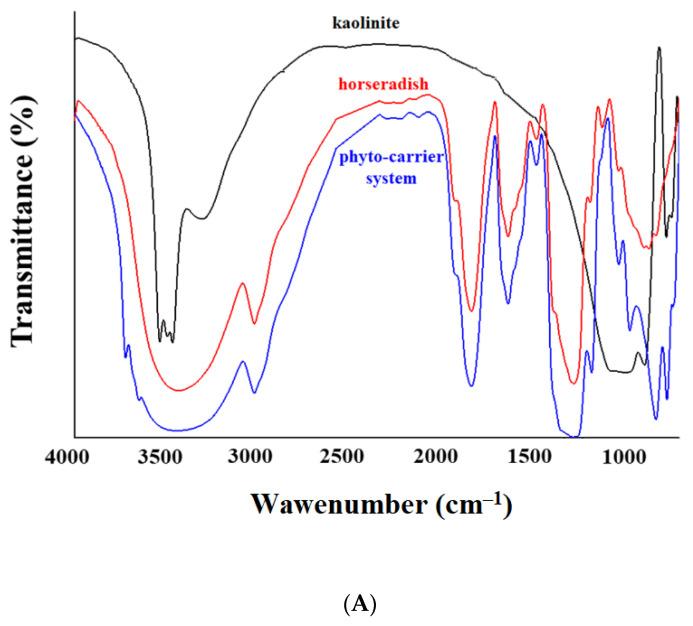

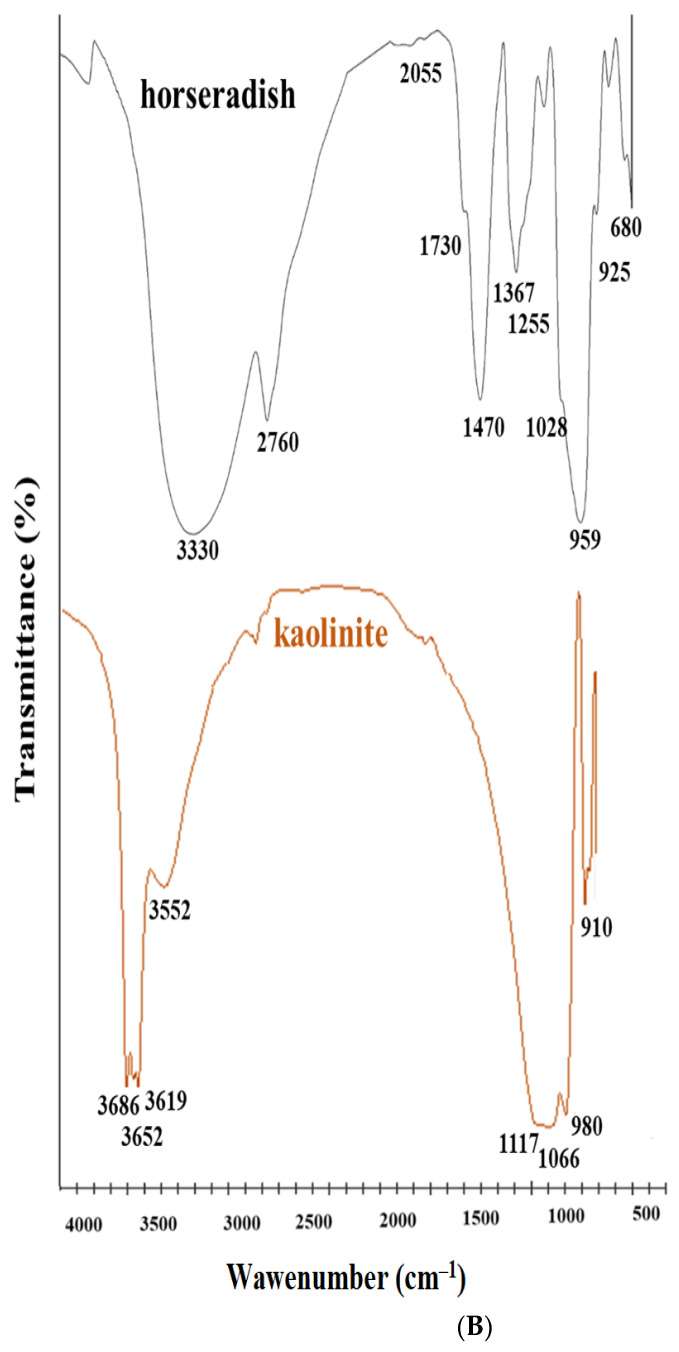
(**A**) FTIR spectra of kaolinite, horseradish, and phyto-carrier system. (**B**) FTIR spectra of FT-IR absorption bands identified in the horseradish sample are presented in the following table (Table 5).

The incorporation of the horseradish phytoconstituents into the pores of the kaolinite particles was successfully achieved and confirmed through FT-IR spectroscopy. Figure 5A presents the spectra of the horseradish, the kaolinite particles, and the new phyto-carrier system.

The FTIR peak of the kaolinite (Figure 5B) presented vibrational bands characteristic at 3686, 3652, 3619, and 3552 cm^−1^ (attributed to the OH stretching vibrations), and 1117, 1066, 980, and 912 cm^−1^ (associated with the Si-O stretching vibration) [100,101,102,103].

The data obtained and presented in Figure 5 confirm the successful development of the new phyto-carrier system.

The obtained IR spectra of the new phyto-carrier system incorporated peaks specific to the secondary metabolites from the horseradish at the following: 3330 cm^−1^, assigned to the OH group; 2760 cm^−1^, attributed to the O-H stretching in the amino acids; 2055 cm^−1^ (N=C=S stretching of isothiocyanate); 1730 cm^−1^ (C-H stretching by methylene groups); 1470 cm^−1^ (C-H bending); 1367 cm^−1^ (O-H bending); 1255 cm^−1^ (C-O stretching); 1028 cm^−1^ (C-N stretching); 959 and 925 cm^−1^ (symmetric N-C-S stretch); and 680 cm^−1^ (aromatic ring); and the characteristic absorption bands of the kaolinite [90,91,92,93,94,95,96,97,98,99,100,101,102,103].

In addition, the kaolinite absorption bands at 3686, 3652, 3619, and 3552 cm^−1^ (attributed to O-H stretching vibrations) and the vibrational bands at 1117, 1066, 980, and 912 cm^−1^ (associated with Si-O stretching vibration) were shifted to lower wavenumbers, indicating that this functional group was involved in the binding of the O-H, C-N, N-H, and C-O functional groups from the horseradish (Figure 5, Table 5) [90,91,92,93,94,95,96,97,98,99,100,101,102,103].

Moreover, several detectable changes occurred in the horseradish spectra, particularly the hydroxyl vibrations (O-H stretching and O-H bending), indicating that this functional group is involved in the binding of kaolinite [23,24,25,26,27,104].

### 3.5. X-ray-Diffraction Spectroscopy

The XRD technique was used to obtain information about the atomic structure of the phyto-carrier system and the raw materials. 

**Figure 6:**
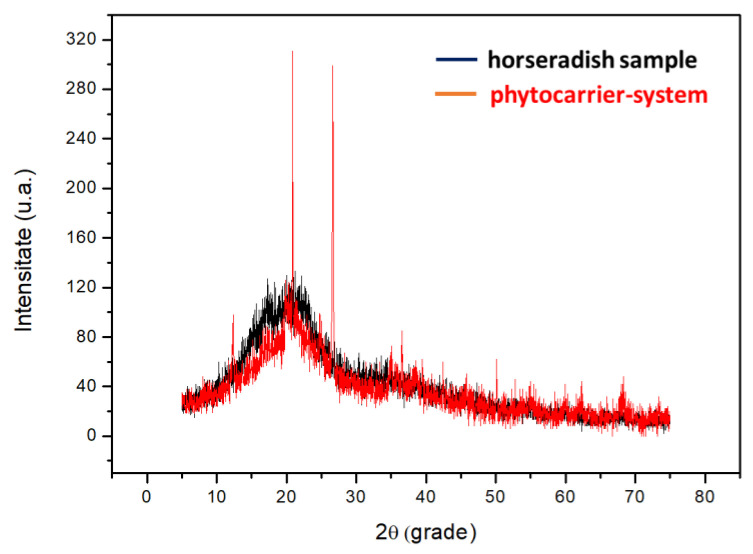
The overlapping XRD spectra of horseradish sample and new phyto-carrier system.

Figure 6 displays the XRD patterns of the horseradish sample and the new phytocarrier system.

In the XRD spectrum of the new phyto-carrier system, the characteristic XRD peaks of the kaolinite and horseradish samples are easily observable. Hence, the absorption peaks at 2θ (degrees) values of 12°, 25°, 34°, 36°, and 51° can be assigned to a triclinic structure [105].

The XRD pattern of the horseradish sample (Figure 6) was in the range of 11.8–34.6°, with large bands and weak peaks characteristic of amorphous phases, which can be attributed to the phytoconstituents from the horseradish (minerals, hydroxides, and fibers).

### 3.6. Scanning-Electron Microscopy–Energy-Dispersive X-ray (SEM–EDX)

Scanning-electron microscopy–energy-dispersive X-ray (SEM–EDX) is a versatile technique to investigate the morphologies, compositions, and microstructures of materials. In some complex materials, it allows the identification of the component phases through qualitative chemical analysis [106].

The morphological changes (the size, shape, and distribution of the particles) in the horseradish and kaolinite samples before and after the preparation of the new phyto-carrier system were investigated by using the SEM–EDX technique.

**Figure 7:**
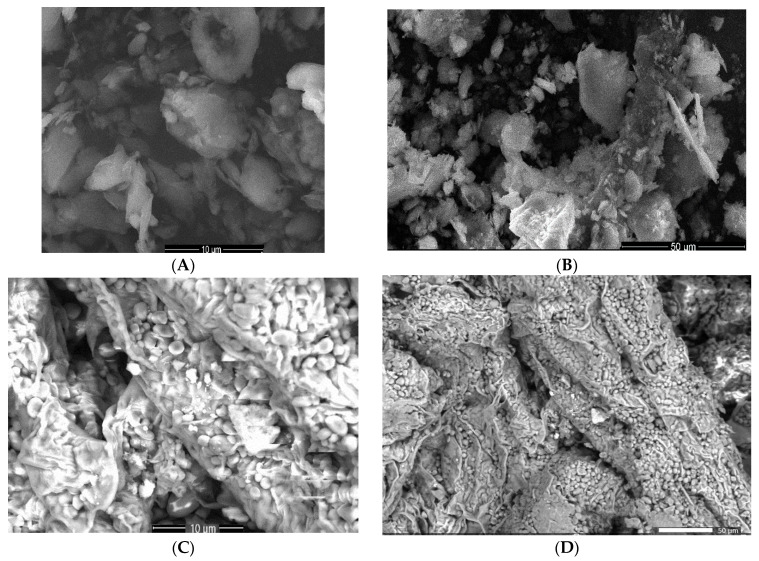

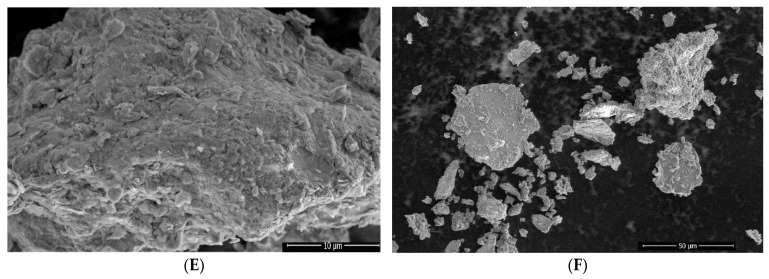
SEM images of kaolinite (**A**,**B**), horseradish (**C**,**D**), and phyto-carrier system (**E**,**F**).

To acquire insights, the SEM micrographs were recorded at different magnifications. The obtained two-dimensional images are shown in Figure 7.

The SEM micrograph of the kaolinite sample (Figure 7A,B) exhibited a heterogeneous size distribution of small anhedral and pseudo-hexagonal particles up to 5 μm in size [25].

It appears that the horseradish micrographs (Figure 7C,D) indicated the presence of a heterogeneous fibrous structure, with a thickness of about a few μm, with porous regions with irregular shapes. These porous regions allowed the arrest of the kaolinite particles.

The morphology of the phyto-carrier system (Figure 7E,F) indicated the presence of kaolinite particles both on the surface and in the porous areas of the horseradish sample. Changes in the sizes of the horseradish and kaolinite particles (reduction) were observed, which can be explained by the experimental conditions of the new phyto-carrier system preparation.

**Figure 8:**
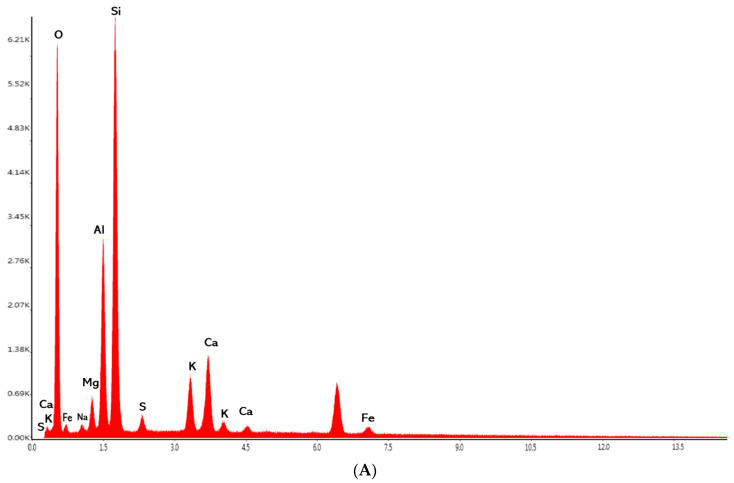

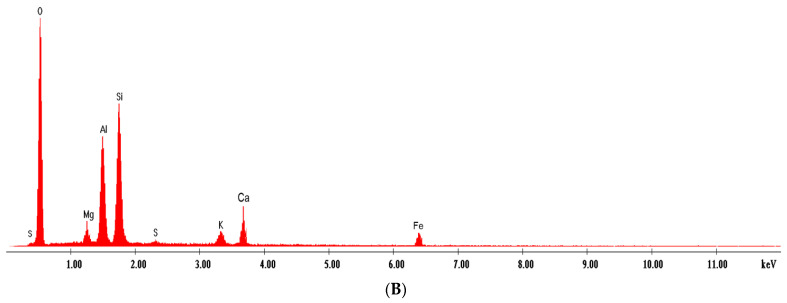
(**A**) EDX composition of kaolinite sample; (**B**) EDX composition of the new phyto-carrier system.

Accompanying the SEM spectra are EDX analyses on the elemental composition of the kaolinite and phyto-carrier investigated (Figure 8A,B).

According to the data from the EDX (Figure 7A), the predominant element contents in the kaolinite sample were silica, aluminum, magnesium, calcium, potassium, iron, sodium, oxygen, and sulphur. Overall, the chemical analysis revealed the significant oxides SiO_2_, Al_2_O_3_, Fe_2_O_3_, MgO, CaO, Na_2_O, K_2_O_5_, and SO^−3^, which was in good agreement with the data reported in the literature [25].

The comparative analysis in Figure 8B highlights the presence of peaks corresponding to the kaolinite (Figure 8A) in the new phyto-carrier system. The EDX results confirmed the preparation of the new phyto-carrier system.

### 3.7. Dynamic Light Scattering (DLS)

Dynamic light scattering (DLS) is a fast and very efficient method for determining the sizes of particles and the particle-size distribution (PSD) in suspensions [107]. Particle-size measurement is established indirectly by using the intensity of the light-scattered fluctuations, yielding the rate of the Brownian motion [107].

**Figure 9:**
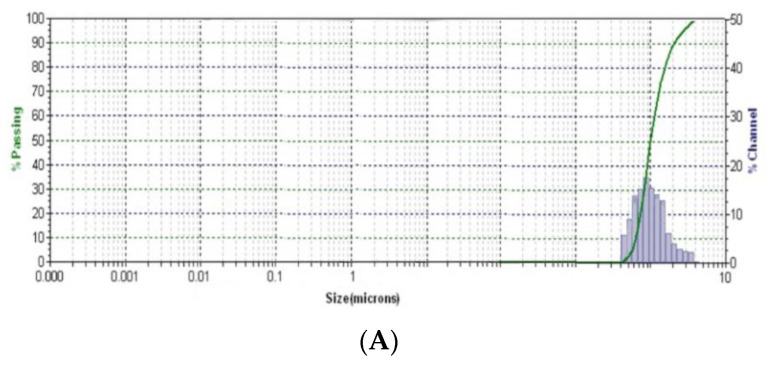

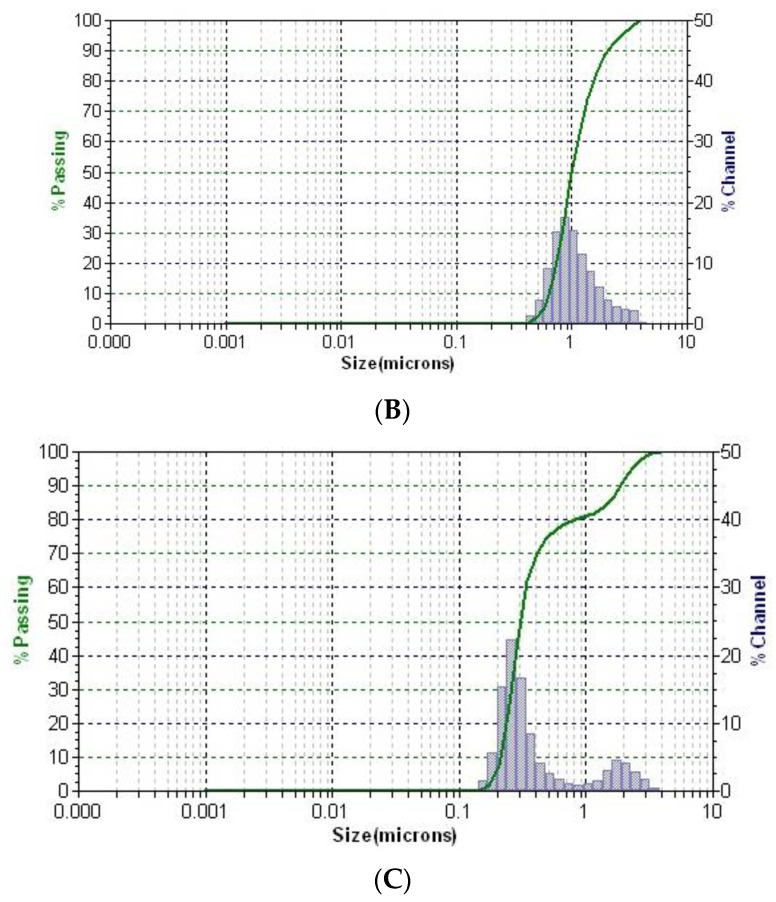
DLS patterns of kaolinite (**A**), horseradish sample (**B**), and phyto-carrier system (**C**).

The DLS method was used to obtain information about the average mean particle size of the phyto-carrier system and its raw components. The DLS results are displayed in Figure 9.

The average diameter size of the kaolinite particles was 500.03 nm (Figure 9A), corroborating the SEM results. In the horseradish sample (Figure 9B), the average diameter of the particles was 100.2 nm.

The DLS pattern of the new phyto-carrier system (Figure 9C) exhibited two peaks that can be attributed to the kaolinite and horseradish particles, distributed in a narrow range. The mean diameter of the kaolinite in the phyto-carrier system was 277.5 nm. The second mean of the hydrodynamic diameter, associated with the horseradish particles, was about 186.4 nm. The fact that the average diameter of the horseradish particles in the phyto-carrier increased compared to that determined in the horseradish sample can be attributed to the loading of the pores on the plant surfaces with the kaolinite particles, which was confirmed by the results of the SEM analysis.

Furthermore, the reduction in the mean size of the kaolinite particles from 500.03 nm (Figure 9A) to 277.5 nm (Figure 9C) was attributed to the experimental conditions for the preparation of the new phyto-carrier. In addition, Figure 9C shows well-dispersed particles of horseradish and kaolinite, which indicates the high stability of the new phyto-carrier system.

### 3.8. Zeta Potential

The zeta-potential method determines the charge of a particle in a suspension, providing an estimation of interactions between particles and the suspension stability.

The zeta-potential value of the kaolinite particles was −35.09 mV, indicating the high stability of the suspension, in good agreement with the data reported in the literature [108].

The zeta potential changed to −23.12 mV for the phyto-carrier system, indicating high biocompatibility.

### 3.9. Screening of Antioxidant Activity

For a specific herb, the total antioxidant capacity (TAC) is the outcome of the cumulative action of entire antioxidant classes from its composition [37]. The adequate investigation of the antioxidant activity of a plant requires an appropriate variety of tests to address the mechanism of action characteristic of each category of phytochemicals [37,38,109,110].

Various chemical (spectrometric, chromatographic, and electrochemical) and biochemical methods have been developed for the assessment of the antioxidant capacities of different biomolecules [37,38,109,110]. The most common are the in vitro tests, divided based on the reaction-mechanism type into hydrogen-atom transfer (HAT) and electron transfer (ET) methods [37,38,109,110].

The first category, HAT methods, includes the oxygen-radical-absorbance capacity (ORAC), the total radical-trapping-antioxidant parameter (TRAP), the total radical-scavenging-capacity assay (TOSCA), the chemiluminescent assay, β-carotene bleaching assays, and the inhibition of induced LDL oxidation [37,38,111,112,113].

The main ET methods (based on electron transfer) are the total phenolics assay (Folin–Ciocalteu reagent assay), the 2,2-Diphenyl-1-picrylhydrazyl radical-scavenging assay (DPPH•), the Trolox equivalence antioxidant-capacity assay (TEAC), the ferric-ion-reducing antioxidant-power assay (FRAP), the cupric reducing antioxidant capacity (CUPRAC) assay, the N,N-Dimethyl-p-phenylenediamine radical-scavenging assay (DMPD•+), and the 2,2-Azinobis 3-ethylbenzthiazoline-6-sulfonic acid radical-scavenging assay (ABTS•+) [37,38,109,110].

The choice of a particular method depends on criteria related to simplicity, sensitivity, associated costs, and reproducibility [37,38,39,111,112].

The biological activity of a plant varies depending on the complexity of the chemical composition and, implicitly, on the collective, complementary, and the synergistic actions of a variety of secondary metabolites. Moreover, the antioxidant activities of plants differ, depending on morphological parts, degree of maturity, and exogenous parameters (temperature, pH, humidity, and others) [37].

Hence, the antioxidant activity of the phyto-carrier system is a combined result of the complementary and synergistic actions of its components (horseradish and kaolinite). A total amount of ninety secondary metabolites from nine different chemical classes were identified in the horseradish sample. Consequently, to consider the antioxidant properties of the new phyto-carrier system more precisely, three different in vitro, non-competitive methods were used (DPPH, Folin–Ciocalteu, and phosphomolybdate (total antioxidant capacity).

#### 3.9.1. DPPH (1,1-diphenyl-2-picrylhydrazyl) Free-Radical-Scavenging Assay

The DPPH (2,2-diphenyl-1-picrylhydrazyl) is a fast, simple, low-cost, and accurate method based on a single electron transfer (ET)-type mechanism for the antioxidant assessment of plant extracts or other complex matrices. Furthermore, it is a highly frequently used assay to determine the free scavenging capacity of antioxidants based on the ability of compounds to act as free-radical scavengers or hydrogen donors [37,38,39,110,111,112,113].

**Figure 10:**
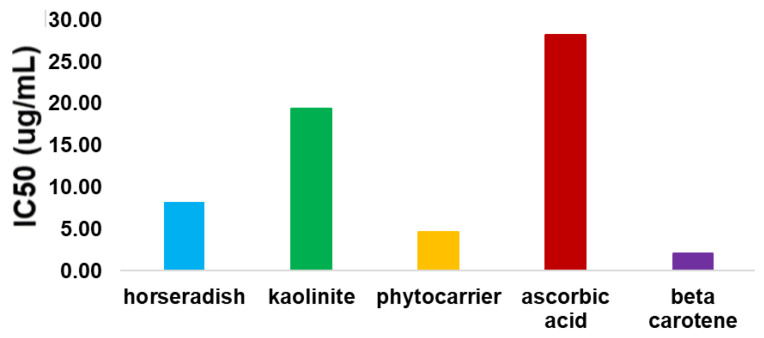
Graphic representation of DPPH results expressed as IC50 (µg/mL).

Hence, the antioxidant activity of the new phyto-carrier system and its components were evaluated in relation to the antioxidant standards of β-carotene and ascorbic acid. It is noteworthy that different studies reported the presence of β-carotene and ascorbic acid in the chemical composition of horseradish [114,115]. The data obtained are presented in Table 6 and Figure 10.

The obtained IC50 values indicated that the antioxidant activity of the new phyto-carrier system was higher than that of the horseradish sample, the kaolinite, and the ascorbic acid. For the new phyto-carrier system, the IC50 value was about half that of the horseradish sample. The increase in the antioxidant activity of the phyto-carrier system compared to the horseradish and kaolinite was in good agreement with the literature data [116,117]. The IC50 value for the beta-carotene standard can be explained by the experimental conditions (the low solubility of beta carotene in methanol) [118].

#### 3.9.2. Folin–Ciocalteu Assay

This assay is widely used as a fast, simple, precise, and inexpensive measure of total phenolics from natural products based on an oxidation/reduction-reaction mechanism (electron transfer) [38,39,41,119,120].

The total polyphenolic contents (TPCs) of the horseradish sample and phyto-carrier system were determined and the obtained results are presented in Table 7.

According to the results, the total polyphenolic content identified in the new phyto-carrier system was more than 39% higher than that of the horseradish sample. The higher antioxidant capacity of the phyto-carrier system compared to the horseradish sample can be attributed to the synergistic action of the kaolinite and corresponds to the data reported in the literature [121].

#### 3.9.3. Phosphomolybdate Assay (Total Antioxidant Capacity)

Phosphomolybdate (total antioxidant capacity) is a frequently used and precise assay used to evaluate the total antioxidant potentials of plant extracts or other complex mixtures of biomolecules. It is based on the Mo(VI)-to-Mo(V) reduction of the presence of antioxidants [44].

**Figure 11:**
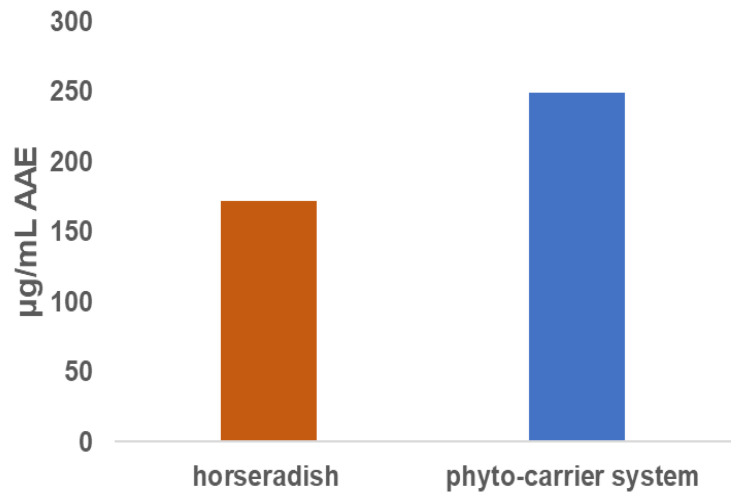
Graphic representation of phosphomolybdate (total antioxidant capacity) results expressed as µg/mL AAE.

The phosphomolybdate assay (total antioxidant capacity) was used to determine the total antioxidant potential of the prepared phyto-carrier system compared to those of the horseradish and ascorbic acid. The obtained experimental results are displayed in Table 8 and Figure 11.

The phyto-carrier system displayed a higher antioxidant activity than the horseradish sample. This result can be attributed to the synergistic and complementary action of the phytoconstituents in the horseradish and the antioxidant mechanism of the kaolinite [28]. In addition, the kaolinite potentiated the antioxidant activities of secondary metabolites in the horseradish sample [121].

## 4. Conclusions

In this study, a new phyto-carrier system with particular morpho-structural properties and high antioxidant activity was prepared. The low-molecular-mass-metabolite profiling and the VOC-aroma profile of the *Armoracia rusticana* grown in the wild in Romania were determined. The biological activities of each identified phytoconstituent category in the horseradish were discussed. The development of the horseradish–kaolinite carrier system was confirmed through FTIR, EDX, XRD, DLS, zeta-potential, and SEM studies. The size distributions of the kaolinite and horseradish particles were investigated through a DSL analysis. The kaolinite and the phyto-carrier system’s stability levels in aqueous suspensions were determined using a zeta-potential analysis. A combination of assays (DPPH, Folin–Ciocalteu, and phosphomolybdate (total antioxidant capacity)) was used to evaluate the antioxidant properties of the proposed phyto-carrier system. The results demonstrated the significantly higher antioxidant activity of the phyto-carrier compared with its components (horseradish and kaolinite). However, further studies are required to investigate the biological activity, bioavailability, and biocompatibility of the new phyto-carrier system. This study may motivate future research on therapies in the area of advanced antitumoral agents.

## Figures and Tables

**Table 3:** Classification of bioactive secondary metabolites from the *Armoracia rusticana* L. sample in different chemical categories.

Chemical Class	Metabolite Name
glucosinolates	glucocapparin
	glucolepidiin
	sinigrin
	gluconapin
	glucocochlearin
	glucobrassicanapin
	glucoconringiin
	glucoiberverin
	glucosativin
	glucotropaeolin
	glucoiberin
	glucoberteroin
	glucocheirolin
	glucobrassicin
	glucoalyssin
	5-hydroxyglucobrassicin
	4-methoxyglucobrassicin
isothiocyanates	allyl isothiocyanate
	3-butenyl isothiocyanate
	isobutyl isothiocyanate
	cyclopentyl isothiocyanate
	2-pentyl isothiocyanate
	benzyl isothiocyanate
	erucin
	phenethyl isothiocyanate
fatty acids	myristic acid
	13-hexadecenoic acid
	beta-hydroxypalmitic acid
	pinolenic acid
	9,10-dihydroxypalmitic acid
	17-hydroxyoleic acid
	14-hydroxystearic acid
	eicosadienoic acid
	10,11-dihydroxy stearic acid
	linoleic acid
amino acids	proline
	threonine
	cysteine
	asparagine
	lysine
	phenylalanine
	arginine
	tyrosine
	proline
phenolic acids	coumarinic acid
	gallic acid
	caffeic acid
	syringic acid
	sinapinic acid
	chlorogenic acid
flavonoids	kaempferol
	catechin
	quercetin
	rutin
terpenoids	α-phellandrene
	menthol
	junipene
coumarins	esculetin
	scopoletin
aldehyde & ketone	pentanal
	3-hexenal
	hexanal
	benzaldehyde
	phenylacetaldehyde
	vinyl amyl ketone
	4-ethylbenzaldehyde
	anisaldehyde
	nonanal
	vanillin
organic acids	acetic acid
	oxalic acid
	isovaleric acid
	γ-aminobutyric acid
	benzyl alcohol
	fumaric acid
	succinic acid
	malic acid
	p-salicylic acid
	ascorbic acid
	citric acid
furans	2-ethylfuran
	2-pentylfuran
miscellaneous	sphingosine
	glucogallin
	allyl cyanide
	naphthalene
	3-phenylpropionitrile
	2-sec-butyl-3 methoxypyrazine

**Table 4:** Aromatic compounds identified in *Armoracia rusticana* using ATOF-MS.

No	Name	Odor
1	acetic acid	vinegar
2	carbonyl sulphide	sulphuric
3	dimethylsulfide	cabbage, sulphurous onion
4	allyl cyanide	onion
5	carbon disulphide	sulphuric
6	pentanal	acrid
7	2-ethylfuran	ethereal rum, cocoa
8	3-hexenal	fruity, green, vegetable
9	allyl isothiocyanate	purgent, sulfuric, mustard, garlic
10	hexanal	green, woody, grassy
11	isovaleric acid	cheesy
12	benzaldehyde	almond
13	benzyl alcohol	floral, berry
14	3-butenyl isothiocyanate	purgent
15	isobutyl isothiocyanate	purgent
16	phenylacetaldehyde	green, floral, honey
17	cysteine	sulphur
18	vinyl amyl ketone	earthy, mushroom
19	naphthalene	mothballs
20	2-pentyl isothiocyanate	purgent
21	malic acid	apple, cherry
22	4-ethylbenzaldehyde	sweet, almond, cherry
23	anisaldehyde	sweet, floral, aniseed
24	α-phellandrene	peppery, woody, grassy
25	p-salicylic acid	phenolic
26	2-pentylfuran	green
27	nonanal	citrus, rose
28	benzyl isothiocyanate	pungent green
29	vanillin	vanilla, sweet
30	menthol	minthy
31	erucin	purgent raddish, cabbage
32	phenethyl isothiocyanate	sulfurous
33	2-sec-butyl-3-methoxypyrazine	bell pepper, galbanum
34	junipene	pine, woody
35	kaempferol	bitter
36	quercetin	bitter
37	glucocapparin	purgent, horseradish-
38	gluconapin	pungent, green, cabagge
39	glucobrassicanapin	acrid, purgent, mustard, horseradish
40	glucobrassicin	purgent
41	glucosativin	rocket
42	glucoiberverin	purgent, radish
43	sinigrin	pungent, sulfurous, mustard
44	glucotropaeolin	purgent

**Table 5:** The characteristic absorption bands attributed to secondary metabolites identified in *Armoracia rusticana*.

Secondary Metabolite	Wavenumber (cm^−1^)	Ref
glucosinolates	990–1090, 1433–1470, 1695, 1730, 1920, 1990–2150, 2270	[91,92]
isothiocyanates	2060–2190. 2269–2275, 1990 − 2150, 2034, 925–1250, 680, 520–570, 425–440, 464	[91,92]
flavonoids	4000–3125, 3140–3000, 1670–1620, 1650–1600, 1600–1500, 1450–1490	[93,94]
amino acids	3400; 3330–3130; 2530–2760; 2130; 1724–1754 1687, 1675, 1663, 1652, 1644, 1632, 1621, 1611, 1500–1600	[95]
terpenoids	2939, 1740, 1651, 810	[96]
phenolic acids	1800–1650, 1734, 1720, 1627, 1522, 1440, 1410, 1420–1300, 1367, 1315, 1255, 1170–1100	[97]
fatty acids	3020–3010, 2924–2915, 2855–2847, 2800–2900, 1746, 1710, 1250, 720	[97,98]
coumarins	2963, 3061, 3381, 1608, 1715, 1489, 1450, 1254, 1028, 600–900	[99]

**Table 6:** IC50 values for horseradish, the new phyto-carrier system, ascorbic acid, and beta-carotene.

Sample Name	Horseradish	Phyto-Carrier System	Ascorbic Acid	Beta-Carotene
IC50 (_g/mL)	8.21.00 ± 0.06	4.68 ± 0.11	28.17 ± 0.02	2.11 ± 0.017

**Table 7:** Total polyphenolic contents in horseradish and the phytocarrier system.

Sample Name	Total Phenolic Content (µg/mL)
horseradish	13.79667
phyto-carrier system	35.18658

**Table 8:** Total antioxidant potentials of phyto-carrier system and horseradish sample.

Sample Name	
horseradish	171.82 ± 0.00343
phyto-carrier system	248.96 ± 0.014

## Data Availability

All data are contained within the article.
